# Exploring Kinematics Information Decoding from EEG Slow Cortical Potentials During Movement Imagination and Observation

**DOI:** 10.3390/s26144456

**Published:** 2026-07-14

**Authors:** Sagila Gangadharan Kutteri, A. P. Vinod

**Affiliations:** Infocomm Technology Cluster, Singapore Institute of Technology, 1 Punggol Coast Road, Singapore 828608, Singapore; vinod.prasad@singaporetech.edu.sg

**Keywords:** brain computer interface, electroencephalogram, motor imagery, kinematics decoding

## Abstract

Motor Imagery-based brain computer interface (MI-BCI) systems capable of decoding imagined movements and their kinematics are a rapidly advancing area of BCI research. Such BCIs can enhance human-computer interaction and have potential neurorehabilitation and assistive technology applications. This study explores the feasibility of decoding kinematic information, including movement direction and speed of imagined hand movements, from EEG slow cortical potentials (SCPs). EEG data from fourteen healthy subjects, associated with bidirectional center-out right-hand movement imaginations at two different speeds, is analyzed in this study. Peak negativity of movement-related cortical potential derived from fifteen primary motor cortex EEG channels is used to decode the direction and speed of imagined and observed hand movements. A Pearson correlation coefficient-based channel selection is further applied to identify a subject-specific set of channels from the pool of fifteen channels for decoding the kinematic information. Pairwise classification of direction-speed combinations achieved an average accuracy of 63.44 ± 9%. In contrast, slow-versus-fast speed classification achieved a lower accuracy of 53.87 ± 6.4% for motor imagery, which was not significantly different from the empirical chance distribution. The same analysis applied to movement observation resulted in an average direction-speed pair classification accuracy of 57.74 ± 8.6%, while speed classification achieved 50.74 ± 8.1%. These findings demonstrate that SCP features contain reliable information related to movement direction, whereas speed-related information appears weaker and less consistent across subjects. The results highlight the potential of SCP-based decoding for directional control and motivate further investigation of speed-related neural signatures. The findings from direction decoding during movement observation open avenues for future investigations into shared neural representations underlying passive movement observation.

## 1. Introduction

Brain-Computer Interface (BCI) is an emerging technology that facilitates a direct communication link between the brain and external devices [1]. It enables the user to achieve control and communication through their brain signal, without involving the regular communication pathway of peripheral nerves and muscles. BCI technology is primarily aimed at providing assistive and rehabilitative support to people suffering from neuromuscular disorders. Motor imagery-based BCI (MI-BCI), which decodes imagined movement from brain signals, has greater potential for providing assistive and rehabilitative support specifically to stroke survivors [2], as it enables movement control through mental imagination, without necessitating actual movement. Early-stage MI-BCI research focused on classifying the imagination of individual body parts like left hand vs. right hand, hand vs. foot, etc., and imagined movement types [3,4,5,6,7]. However, these MI-BCIs offer a limited degree of freedom of movement and hence a limited number of control commands, which restricts their suitability for rehabilitation and neuroprosthetics. Real-world applications require MI-BCI systems that can offer natural, continuous, and precise motor control to external effectors, which can be achieved by designing MI-BCI systems to have a higher degree of freedom of movement that offers a higher number of control commands to externally interfaced devices.

Movement kinematics decoding involves decoding multiple parameters of movement like speed, velocity, direction, force, position, etc. [8], and has gained significant attention recently, as it can offer an increased number of control commands compared to conventional MI-BCIs. Kinematic parameters of executed movements were decoded from non-invasive EEG in many studies. Directions of executed voluntary hand movements of seven healthy subjects were decoded using EEG in [9]. The authors proposed Wavelet Phase Locking Value features to decode movements in four orthogonal directions and achieved a mean binary classification accuracy of 76.85%. Slow- and fast-executed movements of the hand performed in four orthogonal directions were decoded in [10] using EEG. Wavelet Common Spatial features were used to decode the speed of movement, resulting in a mean classification accuracy of 83.71% among seven healthy subjects. The authors also reconstructed the speed profile using multiple linear regression, resulting in a mean Pearson correlation coefficient of 0.52 between reconstructed and recorded speed profiles. Peak speed and acceleration of spatially directed reaching movements were decoded using EEG in [11]. The authors estimated the peak speed and acceleration using EEG alpha (8–12 Hz) and beta (13–30 Hz) power from the movement planning and execution phase and reported that the combination of features from both the planning and execution phases further improved the decoding accuracy. The continuous trajectory of bimanual coordinated movements was decoded from EEG in [12]. The authors decoded bimanual trajectories including velocity and position using a deep learning model combining EEGNet and a Long Short-Term Memory network. Grand average correlation coefficients of 0.54 and 0.42 were achieved among thirteen subjects for position and velocity, respectively. Movement intentions and levels of force and speed of intended movement were decoded from EEG in [13]. The authors performed a classification of movement intentions, varying levels of force, and speed using temporal features derived from movement-related cortical potential.

Decoding the kinematic parameters of imagined movement is more challenging than decoding the kinematics of executed movement. Imagined 3D hand movement trajectories were decoded from EEG in [14]. The authors used an EEG power spectral density-based band power time series for decoding the trajectory of kinesthetically imagined 3D hand movement tasks. The results reported higher reconstruction accuracy (R-value ~0.4) with the band power time-series model with mu (8–12 Hz), beta, and low gamma bands (28–40 Hz) than with the conventional potential time-series model (R-value ~0.15). Continuous decoding of upper-limb imagined trajectories during center-out reaching tasks is attempted in [15]. Imagined trajectories were reconstructed using multidimensional linear regression. Though the decoding performance was poor (success rates of 14.05, 27.67, 29.9, 61.52, and 55.61 for different target configurations), the authors reported a significant correlation coefficient for the horizontal velocity component. Imagined directions of center-out hand movements in two directions were decoded in [16]. The authors reported an average direction classification accuracy of 73.33% across fifteen healthy subjects using envelope and phase features derived from parietal EEG.

Research studies on decoding imagined movement kinematics have primarily focused on extracting individual kinematic parameters, such as movement speed or direction, from non-invasive EEG signals. However, real-world motor tasks generally require the simultaneous modulation of multiple kinematic parameters to achieve the desired movement. Therefore, MI-BCI systems capable of decoding imagined movements involving multiple kinematic dimensions are essential for developing more natural and intuitive control strategies. EEG correlates associated with multi-directional hand movements performed at different speeds have previously been investigated in [10]; however, this study focused on actual movements rather than motor imagery. In [17], movement type and speed of imagined wrist movements were decoded from nine healthy participants. Rebound rate of movement-related cortical potentials (MRCPs), along with mu and beta band power features, were used to classify four right wrist movement tasks performed at two different speeds (slow and fast), achieving an average misclassification rate of 21 ± 2% between task pairs. Motivated by these findings, our previous work [18] investigated the decoding of imagined hand movement kinematics involving two different movement directions and two speeds (slow and fast). Wavelet Common Spatial Pattern (WCSP) features and MRCP-based features were employed to classify direction-speed combinations as well as movement speed. Pairwise, binary classification of direction-speed pairs achieved an average accuracy of 68.11 ± 9.8%, while slow versus fast speed classification achieved an accuracy of 59.52 ± 5.5% across fourteen healthy participants.

The present study extends our previous work [18] beyond decoding performance evaluation by investigating the underlying EEG characteristics associated with movement direction and speed, with particular emphasis on slow cortical potentials (SCPs) during motor imagery. Furthermore, since each experimental trial included a movement observation period preceding motor imagery, this study also investigates whether kinematic-related neural information is represented in EEG during passive movement observation. This investigation is motivated by the mirror neuron theory, discovered in the early 1990s. Mirror neurons represent a distinctive class of neurons that discharge when an individual executes a motor act as well as when they observe another individual performing a similar motor act [19]. The studies on mirror neuron theory have reported that while observing an action, the mirror neurons fire in the same way when we recreate that action ourselves [19,20,21]. Though many EEG-based non-invasive studies were conducted on movement observation, they were mainly focused on investigating the nature of brain activation during different action observations and understanding the encoding mechanism of the brain during action observation [22,23,24,25]. Whether kinematic information related to the observed movement is reflected in EEG signals during passive movement observation remains largely unexplored. A better understanding of EEG-based kinematic decoding during movement observation could provide further insights into shared neural representations between observed and imagined movements and contribute to the development of future BCI applications.

Thus, the contribution of this paper is twofold. Primarily, this study investigates the feasibility of decoding movement direction from EEG slow cortical potentials (SCPs) during imagined bidirectional center-out right-hand movements. Peak negativity features derived from subject-specific selected EEG channels are used to characterize movement-related information and evaluate their potential for decoding imagined movement direction. In addition, the possibility of extracting movement speed information from SCP features is explored as a secondary analysis by considering two different movement speeds (slow and fast).

Secondly, this study explores whether movement-related information is preserved during passive movement observation. The similarity of SCP features between motor imagery and movement observation is examined, and the feasibility of decoding movement direction and speed from observation-related EEG is further evaluated. These analyses provide additional insights into the extent to which SCP-based features capture shared neural representations associated with active imagination and passive observation.

## 2. Materials and Methods

### 2.1. Experimental Paradigm and Data Collection

**Figure 1:**
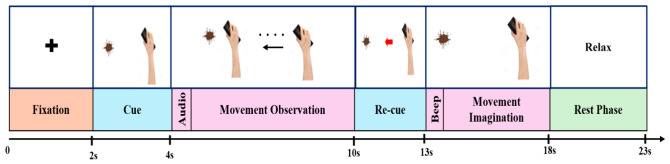
Experimental timeline of a single trial. The arrow and ellipsis indicate that, during this phase of the experiment, the hand image moved from the center toward the direction specified by the arrow.

The EEG dataset [18] used in this study consisted of EEG data from fourteen healthy subjects in the age group 24–38 years (30.9 ± 4.5, mean ± S.D.), including seven male and seven female subjects. Participants were recruited based on the following inclusion criteria: healthy adults with normal or corrected-to-normal vision and no prior history of neurological or psychiatric disorders. Participants were excluded if they had a history of neurological or psychiatric conditions. The detailed participant demography is presented in Table 1. The study comprised all fourteen subjects recruited, and no subjects were excluded after data acquisition. All the subjects were right-handed. The study complies with the Declaration of Helsinki and is performed according to the institute’s ethics committee approval. The details of the experimental protocol were explained to all the subjects before the start of the experiment, and all of them signed written informed consent. The experimental paradigm is designed as a whiteboard cleaning game to facilitate movements in multiple directions at multiple speeds. A Graphical User Interface (GUI) is also developed using MATLAB (v2021) app designer to provide visual cues to the subject during experimental trials. The visual cues involved in the GUI and the experimental timeline for a single trial are shown in Figure 1.

During the experiment, the subjects are seated in a comfortable chair, facing the computer screen, and are instructed to fix their eyes on the center of the screen. Each trial started with a fixation period, during which a fixation cross appeared on the screen. The fixation period lasted for 2 s and was followed by the cue period. During the first visual cue period (2–4 s), a hand image holding a duster appeared at the center of the screen, followed by a blot on either the left or right side of the hand image. This is the direction cue for the imagination task. The position of the blot indicates the direction towards which hand movement imagination is to be performed. The speed of imagery is then indicated by an audio cue. The audio cue indicated the speed of imagination, playing either ‘Move slow’ or ‘Move fast’, to hint to the subject about the movement imagination speed. All the subjects who participated in the study were new to motor imagery, and hence, to help the subjects perform the imagery task correctly, an illustration period was also included in each experimental trial. The illustration period immediately followed the audio cue, and during this period, the hand image moved towards the direction of the blot at a speed indicated by the audio cue and wiped off the blot. This provided the subjects with an illustration of the specific movement to be imagined. The illustration period lasted for 6 s (4–10 s), starting from the onset of the audio cue. Movement of the hand image from the center of the GUI to the position of the blot is programmed to be completed in 4 s for slow movement and 1 s for fast movement. The hand image wiped the blot and then remained still at the final position for the rest of the illustration period. During the illustration period, the subjects are instructed to sit and observe the movement of the hand, without performing any actual movements or movement imagination, and this corresponds to the movement observation period. After the completion of the movement illustration, the cue is displayed again on the screen, along with an arrow indicating the direction of movement. The secondary cue period lasted for 3 s. (10–13 s) and is followed by an audio beep. The audio beep indicates the start of the imagination segment. Upon hearing the beep, the subject has to imagine the movement of his dominant hand toward the direction of the blot at a slow or fast pace, as illustrated before. The imagination period lasted for 5 s (13–18 s of the trial) and is followed by a break period of 5 s. Thus, each experimental trial lasted for 23 s, and every subject completed a total of 48 trials with speed-direction pairs distributed uniformly but in a random manner. EEG was recorded continuously using a 64-channel Brain Vision actiCHamp amplifier (Brain Products GmbH, Gilching, Germany) [26] at a sampling frequency of 500 Hz. EEG is recorded from 35 electrodes over the frontal and parietal regions of the brain, with TP10 as the reference electrode. All the participants in the present study were new to motor imagery, and hence, they were allowed to practice the imagery task during the electrode preparation time, before starting EEG recording. Further, the participants were instructed to avoid unnecessary facial, eye, and body movements during the movement observation and imagery period to minimize artifacts in the recorded EEG. Before analysis, the EEG segments were visually inspected for prominent artifacts, particularly ocular artifacts such as eye blinks and large amplitude fluctuations. EEG trials exhibiting clear artifacts were excluded from further analysis.

### 2.2. Proposed Methodology

**Figure 2:**
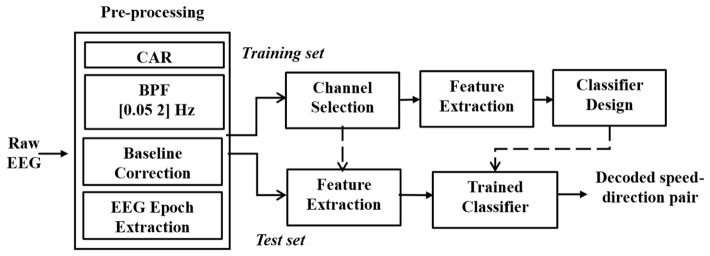
Block diagram of the proposed methodology.

A schematic diagram of the proposed kinematics decoding algorithm is shown in Figure 2. Out of the 35 electrodes, raw EEG data from only 26 electrodes (F3, F1, Fz, F2, F4, FC5, FC3, FC1, FCz, FC2, FC4, FC6, C5, C3, C1, Cz, C2, C4, C6, CP5, CP3, CP1, CPz, CP2, CP4, and CP6) of the primary motor cortex are considered for analysis in the proposed method. These electrodes were chosen based on previous MI-BCI studies [27,28] reporting predominant movement information over sensorimotor regions and help to minimize the influence of horizontal eye movement artifacts on the direction decoding accuracy, as these electrodes are spatially distant from the primary sources of ocular artifacts. Initially, the raw EEG signal is pre-processed to enhance the signal quality. Pre-processing involved Common Average Referencing (CAR), bandpass filtering in the frequency range [0.05–2] Hz, using a second-order zero-phase Butterworth filter and baseline correction. EEG signals were re-referenced using a common average reference calculated across the 26 electrodes selected for motor imagery decoding. The reference montage was restricted to the same electrodes used for MI decoding, to align with the practical requirement of a reduced-channel BCI system. EEG is then filtered into a lower frequency band of [0.05–2] Hz, as the proposed method employs EEG features derived from SCPs to decode the imagined speed-direction kinematics. The chosen bandpass range of 0.05–2 Hz was motivated by prior studies [29,30] reporting that EEG signals below 2 Hz (slow cortical potentials) carry discriminative information for movement-related decoding. Baseline correction was applied by subtracting the mean EEG activity during the 1 s fixation period (1–2 s of the trial) from each trial. Finally, the EEG segments were extracted based on the analysis window of interest. Fifteen electrodes (FC3, FC1, FCz, FC2, FC4, C3, C1, Cz, C2, C4, CP3, CP1, CPz, CP2, and CP4) are considered for extracting SCP features. A discriminative set of channels is identified out of the 15 channels by applying channel selection, and SCP features extracted from the selected channels are then used to decode the imagined kinematics. Binary classification of the speed-direction pair is done using the Support Vector Machine (SVM) classifier. Since the experimental paradigm involved two levels of speed and two directions, it resulted in four speed-direction combinations and hence 6 possible binary classification pairs. All the signal processing was done in MATLAB (v2021).

### 2.3. Subject-Specific Channel Selection

The subject-specificity of discriminative EEG channels in MI-BCIs was investigated previously in several studies [31], which reported improved MI decoding performance with channel selection. In the proposed work, before the extraction of SCP features, a discriminative set of channels is identified from the pool of 15 electrodes based on the Pearson correlation coefficient (PCC). The channel selection algorithm identifies the discriminative set of channels for each binary speed-direction pair as described below.

During the binary classification of each direction-speed pair, for each channel, the SCP signals corresponding to the two classes are trial-averaged to yield two trial-averaged SCP signals corresponding to each class involved in the binary classification. Let $\xi_{i,j}(t)$ represent the SCP signal of the *i*th trial from the *j*th channel. $Z_{c}^{j}\left(t\right)$ is the trial-averaged SCP of the *j*th channel for a given direction-speed pair *c*, and is obtained by averaging the *j*th channel SCPs of all the training trials belonging to direction-speed pair *c*, as in (1):

(1) $$Z_{c}^{j}\left(t\right)=\frac{1}{T_{c}}\sum_{i\in C}\xi_{i,j}(t)\;\;i=1,2,\ldots ,\;n_{t},\;j=1,2,\ldots ,15$$

where $n_{t}$ represents the total number of training trials and $T_{c}$ represents the number of trials belonging to class *c*, where C∈{left−slow, left−fast, right−slow, right−fast}. The trial-averaged SCPs of both of the direction-speed classes $Z_{c1}^{j}\left(t\right)$ and $Z_{c2}^{j}\left(t\right)$ are concatenated together to yield a single vector $Z^{j}\left(t\right)$ where $c_{1}$ and $c_{2}$ represent the two direction-speed classes considered in the binary classification. Thus, $Z^{j}\left(t\right)$ is the concatenated SCP vector for the *j*th channel, having the first half of its samples representing one class and the other half representing the second class in the binary classification. PCC is calculated between the resulting SCP vector $Z^{j}\left(t\right)$ and label vector *L*. Label vector *L* is obtained by concatenating a vector of 1 s and a vector of −1 s, each of length *N*, where *N* is the length of an SCP signal. The PCC value for the *j*th channel is calculated using (2) and represents the correlation between the trial-averaged SCP of each class and the class labels:

(2) $$\rho^{j}=PCC\left\{Z^{j},L\right\}$$

PCC is evaluated for all 15 channels and is considered the discriminant metric for channel selection. To identify the discriminative set of channels, all 15 channels are ranked in decreasing order of absolute value of PCC, and the top-ranked *K* channels are identified as the discriminative channel set for the specific binary speed-direction pair. Only the training data in each fold is used to rank the channels, keeping the test data unseen. The value of *K* is chosen in a subject-specific manner through nested cross-validation, i.e., within each training fold, an inner loop selects the best *K*, which is then applied to the unseen test data. However, the peak value of subject-specific channel size was limited to 10 to limit the feature dimension.

### 2.4. SCP Feature Extraction and Classification

Movement-related cortical potential (MRCP) [32], a slow cortical potential recorded from the centroparietal regions of the brain before and at the onset of voluntary movements, was used widely in motor BCIs for decoding movements and movement kinematics. MRCP is a low-frequency negative shift in the EEG, reflecting the cortical process involved in movement planning and movement preparation [33]. MRCP is recorded in the frequency range [0.05–3] Hz, and MRCP features like peak negativity, peak negativity latency, rebound rate, etc., are reported to be effective in decoding imagined and executed movements and their kinematics [13,17,34,35,36]. Thus, in the proposed work, the peak negativity of the SCP ([0.05–2] Hz) is used as a feature to decode the direction and speed of imagined hand movement. SCP features are extracted from the selected channels, and an SVM classifier is used to decode the binary direction-speed pairs. MATLAB’s Statistics and Machine Learning Toolbox is used to fit the classifier model and to evaluate the decoding accuracy. The subject-specific channel selection and classification were evaluated using nested 4-fold cross-validation, and the classification accuracies reported were obtained by averaging the accuracies of all the folds.

### 2.5. Kinematics Decoding of Observed Movement

In addition to decoding the direction and speed associated with imagined movement, this study also attempted to decode the direction and speed parameters associated with movement observation. Towards this, the performance of the proposed direction-speed decoding algorithm is evaluated for both the motor imagery EEG segment and the EEG segment during movement observation. In each trial, the EEG segment during 4–10 s corresponds to movement observation, and the EEG segment during 13–18 s of the trial corresponds to movement imagery. Five-second-long EEG segments (4–9 s) of the movement observation block and 5 s EEG segments during MI are considered for evaluating the performance of the proposed algorithm. Though the movement observation period ranged from 4 to 10 s of the experimental trial, only the first 5 s (4–9 s) of this period are considered for analysis so that the EEG segments corresponding to movement imagery and movement observation are of identical length. In addition to the comparison of kinematics decoding performance, the EEG patterns associated with motor imagery and movement observation are also analyzed to explore whether the signal patterns elicited during movement imagery and movement observation exhibit any similarity, which is then quantified using the Pearson correlation coefficient.

## 3. Results

### 3.1. Slow Cortical Potential During Motor Imagery

**Figure 3:**
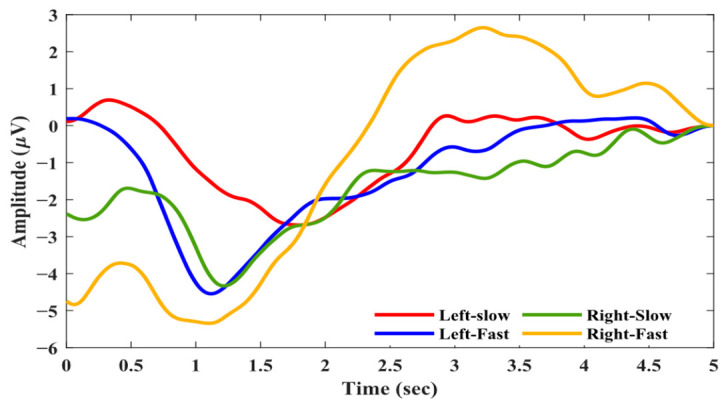
Trial-averaged slow cortical potential at Cz electrode during MI-subject average.

**Figure A1:**
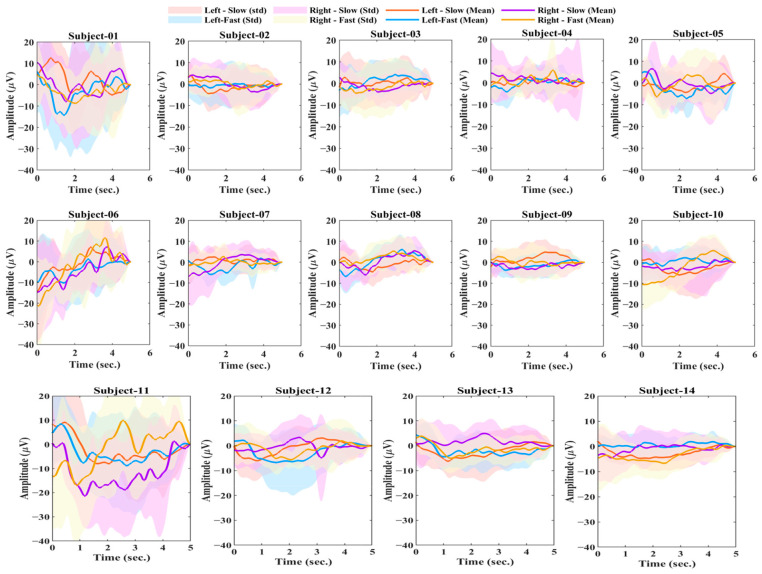
Trial-averaged SCP waveforms of all fourteen subjects during movement imagination.

The trial-averaged SCPs averaged across all fourteen subjects during imagined bidirectional hand movement at slow and fast speeds are shown in Figure 3, corresponding to a 5 s-long imagery segment, recorded from a representative electrode, Cz. The subjectwise trial-averaged SCP waveforms during movement imagery are shown in Figure A1. The curves represent the temporal variation in the EEG slow cortical potential during imagination of hand movement, in a specific direction (right/left) at a specific speed (slow/fast), starting from the onset (t = 0) of the imagination segment. However, it should be noted that the onset of the imagery segment and the actual onset of movement imagination need not be identical and can occur at variable latency. From Figure A1, it can be observed that the trial-averaged SCP patterns exhibit discriminability between the different direction and speed pairs, though it is not consistent in all subjects. This suggests inter-subject variability of the SCP patterns, associated with MI of the right hand in different directions at different speeds. However, a negative shift in the SCP is evident in the majority of the subjects, with a varied trend with respect to the subjects and specific direction-speed pairs. For example, the trial-averaged SCP of Subject-1 shows a clear negative shift in EEG for all the direction-speed pairs. However, it can also be noted that the peak value of this negative shift and the time of occurrence of the negative peak vary with different direction-speed pairs.

**Figure 4:**
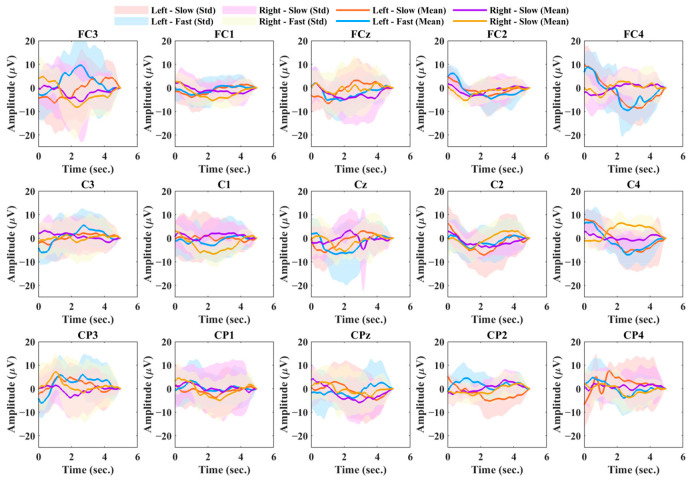
Trial-averaged slow cortical potential of Subject-12 recorded from different electrodes.

Furthermore, to explore the intra-subject variability of SCP patterns, trial-averaged SCP patterns of a representative subject (Subject-12), corresponding to different channels, are illustrated in Figure 4. Trial-averaged SCPs are plotted for 15 electrodes (FC3, FC1, FCz, FC2, FC4, C3, C1, Cz, C2, C4, CP3, CP1, CPz, CP2, and CP4) of the sensory-motor region of the brain, which are considered for extracting SCP features in the proposed work. The channel arrangement follows the approximate sensorimotor scalp topography, with FC, C, and CP electrodes presented in individual rows. From Figure 4, it can be observed that though the negative shift in EEG is evident in all the channels, the temporal variation in SCP, and hence the discrimination offered by SCP between the direction and speed pairs, varies across the channels. This suggests that the SCP patterns associated with multi-directional multi-speed MI exhibit intra-subject variability as well as inter-subject variability. These trends and dependencies exhibited by the SCP patterns are exploited to improve the kinematics decoding performance in the proposed work by considering subject-specific channels for SCP feature extraction.

**Figure 5:**
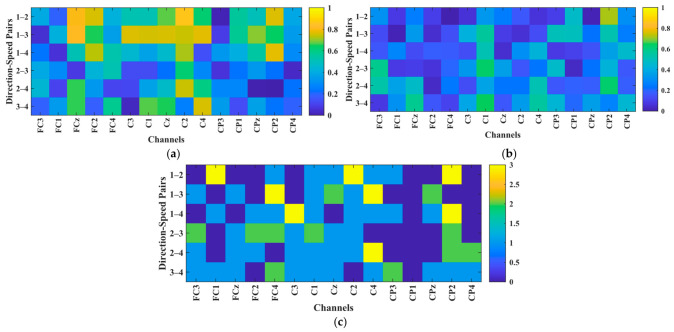
Distribution of the absolute value of the Pearson correlation coefficient across channels and direction-speed pairs: (**a**) Subject-9; (**b**) Subject-12; and (**c**) frequency map showing how often each channel is ranked as top among all subjects for different direction speed pairs.

The subject-specific set of channels for SCP feature extraction is identified using the Pearson correlation coefficient-based channel selection algorithm. The topographical distribution of the absolute value of PCC for two representative subjects is shown in Figure 5. Figure 5a,b shows the PCC distribution for subject-09 and Subject-12, respectively. The channel labels are given along the x-axis and different direction-speed pairs along the y-axis. The direction-speed pairs left-slow, left-fast, right-slow, and right-fast are indicated by group numbers 1, 2, 3, and 4, respectively. The absolute value of PCC exhibited by a specific channel for a given speed-direction pair is represented by each entry, using the color scale ranging from 0 to 1. A higher value of PCC indicates higher discriminating capability offered by the SCP of the specific channel between the given speed and direction pairs. It can be observed that, for a given subject, the channels offering higher values of PCC are not identical for different direction-speed pairs. From Figure 5a,b, it is also evident that the channels of higher PCC also exhibit variability across subjects. Figure 5c shows a frequency map indicating how often each channel was ranked as the top channel across the fourteen subjects for different classification conditions. The frequency map further confirms that no single channel was consistently ranked as the top channel across all subjects and direction-speed conditions. Thus, the channels providing higher discriminability, as well as the number of discriminative channels, varied across subjects and, within each subject, across different direction-speed pairs. These observations justify the subject-specific and direction-speed pair-specific selection of discriminative channels employed in the proposed methodology.

### 3.2. Decoding Directions and Speeds Using MI EEG

The experimental paradigm in the present study involved two directions (right/left) and two speeds (slow/fast), resulting in four direction-speed pairs. Binary classification of the direction-speed pairs is done using the SCP features extracted from the selected set of channels. In addition to the binary classification of the direction-speed pairs, classification of imagined speed is also performed by considering the speed parameter alone, ignoring the direction component. Kinematics decoding is performed using the SCP features, namely mean SCP, peak negativity, and peak latency, extracted from the motor imagery EEG segment. The decoding performance of the individual SCP features is evaluated by carrying out the direction-speed classification using individual SCP features and their combination. The resulting average direction-speed classification accuracy and average slow vs. fast speed classification accuracy are given in Table 2. It should be noted that the average slow vs. fast accuracy given in Table 2 is the average speed classification accuracy across all the subjects, and the values indicated for direction-speed classification are the average value of the resulting mean accuracy of six possible direction-speed binary classification accuracies.

From Table 2, it can be observed that the highest direction-speed classification accuracy is obtained when peak negativity of SCP is used as the discriminative EEG feature for classification. Peak negativity offered higher direction-speed classification accuracy than that offered by other individual features and their combination. Thus, peak negativity is considered the feature for subsequent analyses. The resulting binary classification accuracies for different direction-speed pairs are given in Table 3. The proposed decoding algorithm employing the peak negativity feature resulted in an average direction-speed classification accuracy of 63.44 ± 9% and slow vs. fast speed classification accuracy of 53.87 ± 6.4% for the motor imagery EEG segment. Further, the Kruskal–Wallis statistical test is conducted to examine whether the peak negativity SCP feature employed in the proposed method offers a statistically significant difference between the different direction and speed pairs. The statistical test revealed a significant difference between the SCP features associated with movement imagination in the right and left directions for various speed conditions. However, SCP features associated with slow and fast movement in the same direction exhibited no significant difference.

### 3.3. Kinematics Decoding During Movement Observation

The direction-speed decoding capabilities of EEG recorded during movement observation are also investigated in this work. The direction and speed classification accuracy are evaluated by applying the proposed algorithm to the EEG segment corresponding to movement observation. EEG segment during movement observation (4–9 s of the experimental trial) is used to investigate the effectiveness of movement observation EEG in decoding observed movement kinematics. The resulting average classification accuracies across fourteen subjects, for binary direction-speed classification and slow vs. fast speed classification, are given in Table 3. The proposed algorithm resulted in an average direction-speed classification accuracy of 63.44 ± 9% using peak negativity features from ~6 channels on average, with MI EEG segment, whereas an average direction-speed classification accuracy of 57.74 ± 8.6% is obtained with movement observation EEG, using peak negativity features from five channels on average. Similarly, for the classification of speed (slow vs. fast), MI EEG yielded an average accuracy of 53.87 ± 6.4%, whereas movement observation EEG yielded a slow vs. fast classification accuracy of 50.74 ± 8.1%. The resulting classification accuracies of MI EEG and movement observation EEG were then tested for statistically significant differences using the Wilcoxon signed rank test. As multiple comparisons are performed, false discovery rate (FDR) correction (Benjamini–Hochberg procedure) is applied, and the corrected *p*-values of the statistical test are also given in Table 3. The results revealed no statistically significant difference between the MI and OB results for all classification conditions. Further permutation testing was conducted to determine the empirical chance level and statistical significance of the decoding performance. The results revealed considerable inter-subject variability in decoding performance. While a larger proportion of subjects demonstrated significant above-chance decoding performance for direction-based classification, particularly for comparisons involving different movement directions, speed classification showed limited statistical significance. These findings suggest that SCPs during motor imagery and movement observation contain more robust information related to movement direction, whereas speed-related information appears weaker and less consistently represented across subjects.

### 3.4. SCP During Movement Observation and Imagery

**Figure 6:**
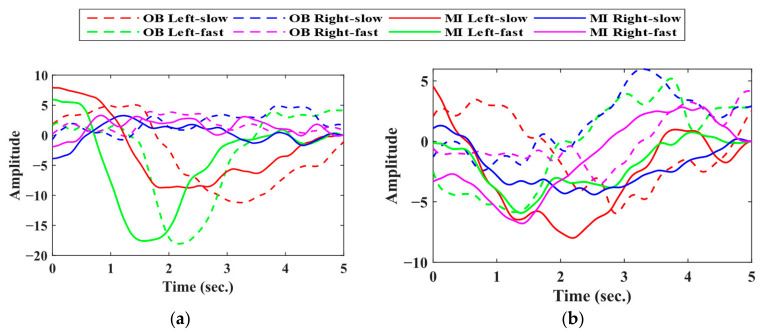
Trial-averaged SCP during movement observation and imagination of (**a**) Subject-8 and (**b**) Subject-12.

As the classification accuracies offered by EEG segments during movement imagery and observation are not significantly different, the SCP patterns during movement observation are evaluated to check for any similarity with those during movement imagery. Trial-averaged SCP patterns during movement observation and imagery are compared to understand whether there is any similarity in the SCPs elicited during movement imagination and observation, as reported in the mirror neuron theory studies [20]. Trial-averaged SCPs of two representative subjects (Subject-8 and Subject-12), during imagination and observation of bidirectional hand movement in specific directions and speeds, are shown in Figure 6. Figure 6a shows the trial-averaged SCP of Subject-8 recorded from the FC4 electrode, and Figure 6b shows the trial-averaged SCP of Subject-12 recorded from the C2 electrode. The prefix ‘OB’ is used in legends to indicate SCPs associated with movement observation (e.g., ‘OB left-slow’) and the prefix ‘MI’ to indicate SCPs associated with movement imagination (e.g., ‘MI left-slow’). From Figure 6a, it can be observed that the trial-averaged SCPs associated with imagined and observed movements are not identical; however, they exhibit a significant amount of similarity and correlation. A similar trend can be observed in Figure 6b as well, with left-slow and right-fast movements. This indicates the similarity between the SCP patterns elicited during movement observation and imagery.

The amount of similarity between the SCPs elicited during observation and imagination of identical movement tasks is quantified using the Pearson correlation coefficient (PCC). PCC is evaluated between the trial-averaged SCPs associated with observation and imagination for each direction-speed pair, for all the 15 electrodes considered for SCP feature extraction. For each subject, the peak value of PCC and the corresponding channel determined for each direction-speed pair are given in Table 4. From Table 4, it can be observed that, except for Subject-6, Subject-7, and Subject-11, all other subjects had peak PCC greater than 0.5 for all the direction-speed pairs, indicating a consistently higher correlation between the MI and OB EEG segments. Subject-6, Subject-7, and Subject-11 exhibited lower values of PCC for right-slow movement. It can also be observed that the channels exhibiting the highest PCC varied between the subjects and with respect to the direction-speed pair for the same subject.

**Figure 7:**
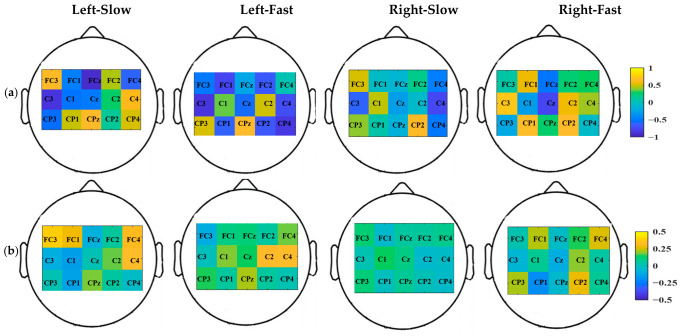
Topographic plot shows the variation in Pearson correlation coefficient between SCPs during movement imagery and observation across channels for (**a**) a representative subject (Subject-12) and (**b**) the subject average.

A topographical plot displaying the variation in PCC with respect to the channels is shown in Figure 7. Each cell with the channel name represents the respective channel, and the value of PCC for the given channel is indicated by the colormap. Figure 7a shows the topographic plot for a representative subject, and Figure 7b shows the topographic plot representing the average PCC across all subjects. It can be observed that for a given direction-speed pair, the channels offer varying amounts of correlation. Further, the topographic pattern of PCC distribution across channels is not the same for all direction-speed pairs.

The SCPs associated with movement observation and imagination are also tested for statistical significance. For each subject, SCP mean values are calculated for each trial, for both the mirror EEG segment and the MI EEG segment. Wilcoxon signed-rank test is conducted on these SCP mean values to check whether the difference between the SCP mean values associated with movement observation and imagery is significantly different. The difference between the SCP means of mirror EEG and MI EEG was not significantly different for all the subjects, for the majority of the channels.

### 3.5. Performance Comparison

The classification accuracies obtained using the proposed algorithm are compared with our previous work [18] on the same dataset, which employed Wavelet CSP and MRCP-based features for decoding imagined movement direction and speed. In [18], motor imagery segments along with the preceding 2 s cue period were employed for decoding analysis. To ensure a fair comparison, the framework reported in [18] was reimplemented by following the original methodology, except for the EEG input segment used for analysis. In the present comparison, the WCSP + MRCP method was evaluated using only the 5 s MI EEG segment to match the analysis window used in the proposed method. Additionally, the same framework was applied to the 5 s movement observation EEG segments to evaluate its performance under the observation conditions. The resulting classification accuracies are presented in Table 5. The WCSP + MRCP approach achieved numerically higher accuracies compared with the proposed SCP-based approach; however, the differences were not statistically significant (all *p* > 0.05), as assessed using the Wilcoxon signed-rank test. These results indicate that the proposed approach provides comparable decoding performance while enabling investigation of SCP characteristics during movement imagery and observation.

## 4. Discussion

In this work, we investigated the feasibility of decoding imagined hand movement kinematics using EEG-derived slow cortical potentials (SCPs). Specifically, the study examined the representation of movement direction and speed information during motor imagery and evaluated the feasibility of decoding multiple kinematic parameters from SCP features. In addition, the study explored whether kinematic-related information is represented in EEG during movement observation and investigated the similarity between EEG patterns elicited during observation and imagination of the same movement tasks involving multiple direction and speed parameters.

The study of the trial-averaged SCP patterns during movement imagination in the right and left direction at two different speeds revealed that the SCPs exhibited temporal patterns that were dependent on the imagined movement kinematics. In general, a negative shift in signal amplitude is observed, the signal amplitude then increases after reaching the negative peak. However, the amplitude of the negative peak and corresponding time index varied according to the direction and speed of the imagery. For example, the trial-averaged SCPs of Subject-1 in Figure A1 show the occurrence of the negative peak at different time indices for slow and fast movements. For a given direction, SCPs of fast movements attained peak negativity at an earlier time index than slow movements. Similar observations were reported for movement-related cortical potentials associated with slow and fast movements in previous studies [36]. However, the SCP patterns exhibited subject-dependent variability, and hence, the patterns were not consistent and identical among all the subjects. In addition to the intrinsic inter- and intra-subject variability associated with non-stationary EEG signals, variable factors involved in performing the imagery task could have also affected the SCP patterns. Though the start of imagery was indicated by an audio beep, the actual onset of imagery can occur at a latency with respect to the onset of the audio beep, from trial to trial and from subject to subject. Moreover, the definition of slow and fast movement can vary from subject to subject as well as from trial to trial, i.e., the exact speed of two slow movements or two fast movements need not be identical for two subjects as well as for different trials of the same subject. It depends on how accurately a subject can reproduce the same imagination during each trial. Subjects may exhibit variability in the onset of motor imagery, the time taken to complete motor imagery, and the speed of imagery. These are inherent challenges in any motor imagery experiment paradigm. These parameters will affect the nature of the SCP patterns in subjects and can be the reason for the variability in the SCP patterns from subject to subject. Furthermore, considering these variabilities, extracting the peak negativity of the SCP from a fixed MI EEG window may inadvertently capture excursions unrelated to movement. This represents a limitation of the current feature extraction approach.

Similar to SCP variability from subject to subject, SCP patterns also exhibited variability between different channels for a given subject. As the SCP indicates the electrical activity of the brain underlying the imagery task, it is obvious that SCP can exhibit variations based on the spatial locations of the brain, reflecting the underlying neurophysiology. The subject- and channel-dependent variability in SCP patterns and trends exhibited by the SCP patterns with respect to imagined direction and speed are exploited in the proposed work to decode the direction and speed of imagined movement. An SCP feature, namely, peak negativity, is considered a discriminative feature to decode imagined kinematics. The subject and channel variability are addressed by proposing subject-specific channel selection for feature extraction using Pearson correlation coefficient-based channel selection. The distribution of PCC shown in Figure 5 also demonstrated subject-dependent variability in channels offering the highest values of PCC. The set of channels offering higher PCC values also varied depending on the imagined speed-direction parameters. These observations on PCC distribution are in concordance with the channel-dependent variability observed in SCP patterns shown in Figure 4, suggesting that channel selection is to be done to identify discriminative channels.

The proposed algorithm using subject-specific channels resulted in a mean direction-speed classification accuracy of 63.44 ± 9% and slow vs. fast speed classification accuracy of 53.87 ± 6.4% using peak negativity of the SCP feature derived from the MI EEG segment. The classification accuracy associated with speed is always less than that of direction-speed classification, and speed classification showed limited statistical significance when evaluated with a permutation test. During speed classification, the classifier is trained using SCP features associated with slow and fast speed, ignoring the direction aspect, which could have resulted in difficulty in learning generalized patterns of speed, as the SCPs corresponding to a given speed also embed patterns associated with multiple directions. Similarly, it is also found that the slow vs. fast classification accuracy for a given direction is lower compared to the right vs. left classification accuracy for a given speed. A possible explanation for this reduced classification accuracy of slow and fast movements in the same direction compared to right and left direction movements at the same speed can be made based on our observation of the statistical test results. The difference in SCP patterns associated with slow and fast movements in a given direction is not statistically significant, whereas the difference in SCPs associated with movements in the right and left direction for a given speed is statistically significant. Hence, it is obvious that as the SCP patterns associated with slow and fast movements in the same direction are not significantly different, the classification of the same will result in lower classification accuracy. Similarly, right and left direction movements at different speeds exhibited statistically significant differences in their associated SCPs, resulting in higher classification accuracy. These observations also pose a new question on the amount of information embedded in the SCPs with respect to individual kinematic parameters, i.e., whether SCP embeds more information on direction than speed, resulting in higher discrimination between two directions than between two speeds. Further, this also indicates that the subjects are not able to elicit statistically different SCP patterns for slow and fast movements. This can be owing to the lack of vivid motor imagery or the inability of the experimental paradigm design to elicit sufficiently distinct neural patterns for slow and fast imagery, resulting in weaker differentiation between the two speed conditions. However, investigating and addressing these questions is beyond the scope of this article and can be addressed in extended future work.

In this paper, in addition to decoding the kinematics of imagined movements, we also investigated the kinematic decoding performance of EEG segments recorded during movement observation. EEG segments obtained during movement observation demonstrated above-chance-level decoding performance for the direction parameter, while speed decoding performance was weak. Furthermore, the similarity between EEG patterns associated with movement observation and imagination was examined and quantified using the Pearson correlation coefficient to explore the potential relationship between observation- and imagery-related neural patterns. The SCP patterns during movement observation exhibited correlations with those during motor imagery; however, these correlations were not consistently observed across all channels. The correlation coefficients showed a non-uniform spatial distribution across channels and varied for different kinematic conditions. Additionally, no clear contralateral dominance was observed among the channels exhibiting higher correlations. Overall, the observed similarity between SCP patterns during movement observation and imagination suggests the presence of partially shared neural representations associated with the same movement tasks, while further investigations with controlled experimental paradigms are required to better characterize these relationships.

The primary objective of including the movement observation segment in the experimental paradigm is to provide visual illustration to aid motor imagery and not for the comparison of SCP patterns between movement imagery and movement observation. Thus, in the proposed experimental paradigm, both the movement observation and imagery segments are included in the same trial with a separation interval of 3 s. Further, both movement observation and imagery periods are preceded by a cue period presenting identical direction and speed cues. This temporal proximity and identical cues may induce shared cognitive preparation, visual entrainment, and expectancy effects, making it difficult to conclude whether the movement-observation decoding truly reflects mirror-related processes rather than carryover from task structure. The direction-speed cue preceding the movement observation window also introduces the possibility of movement preparation for the subsequent motor imagery task, rather than purely reflecting the observation of the movement illustration on the screen. Therefore, this preparatory activity may have contributed to the decoding accuracies obtained during movement observation. In addition, as the visual stimulus presented on the screen varied across different direction-speed conditions, these differences in visual input may also have contributed to the observed decoding accuracy during movement observation. Hence, the findings presented in this paper regarding the similarity between SCPs associated with movement imagery and observation, as well as the decoding potential of movement observation EEG for movement kinematics, cannot be considered definitive. To validate the findings and to derive conclusive evidence on the similarity of SCPs of movement imagery and observation, an experimental paradigm should be designed such that movement observation and imagery tasks are presented in separate trials with randomized breaks between them to ensure separation between the effects of movement imagery and observation. Such a controlled design would also enable a more rigorous evaluation of the decoding potential of movement-observation EEG. Our future work will incorporate these control conditions to disentangle mirror-related activity from expectancy effects and to further validate the kinematic decoding potential of movement observation EEG.

To minimize the potential influence of ocular artifacts, feature extraction and classification were restricted to sensorimotor electrodes, which are spatially distant from the major sources of horizontal eye movement artifacts. Additionally, EEG segments were manually inspected to exclude visible blink artifacts. However, these measures cannot completely eliminate ocular contamination, and this is a limitation of the current work. Future studies will incorporate Independent Component Analysis-based artifact correction to further confirm the robustness of SCP-based direction decoding.

The study presented in this paper is conducted on a limited number of 14 healthy subjects with only 48 trials, limiting the scope of the results. This small sample size can affect the statistical power and generalizability of the findings. Hence, findings should be regarded as preliminary and hypothesis-generating rather than definitive. Further, the subjects who participated in this study are novices in motor imagery and did not receive any prior motor imagery training, except for the short practice session just before the start of the experiment. The lack of prior motor imagery training and measures to rate the vividness of motor imagery are limitations of the present study, which might have resulted in intra-subject variability in SCP patterns and reduced discriminability between different imagined speed conditions. Future studies with larger cohorts are necessary to validate the preliminary findings and to strengthen their generalizability. Thus, future studies will focus on optimizing the experimental paradigm to enhance the separability of EEG patterns associated with different movement directions and speeds. In addition, controlled experimental designs incorporating separate observation and imagery conditions will be explored to further investigate the relationship between neural representations during movement observation and motor imagery.

## 5. Conclusions

In this work, we investigated the feasibility of decoding movement-related kinematic information from EEG slow cortical potentials (SCPs) during imagined and observed right-hand movements in fourteen healthy subjects. The proposed approach demonstrated the potential of SCP features in decoding movement direction, achieving average direction-speed pair classification accuracies of 63.44 ± 9% and 57.74 ± 8.6% during motor imagery and observation, respectively. In contrast, speed decoding showed lower and less consistent performance, with accuracies of 53.87 ± 6.4% and 50.74 ± 8.1% for movement imagery and observation, respectively. These findings suggest that SCPs provide reliable information for movement direction decoding, while speed-related information remains challenging and requires further investigation. Further, the study explored the similarity of SCP patterns between movement imagery and observation EEG. The observed correlations provide preliminary evidence of shared movement-related neural representations between imagined and observed movements; however, the current experimental design and methodological limitations warrant further validation through controlled future studies.

## Figures and Tables

**Table 1:** Demographic details of participants.

Subject ID	Age	Sex	Handedness	Prior BCI Experience
S01	29	M	Right	Nil
S02	37	M	Right	Nil
S03	29	F	Right	Nil
S04	27	F	Right	Nil
S05	31	F	Right	Nil
S06	24	F	Right	Nil
S07	28	M	Right	Nil
S08	36	M	Right	Nil
S09	33	F	Right	Nil
S10	32	F	Right	Nil
S11	30	F	Right	Nil
S12	35	M	Right	Nil
S13	38	M	Right	Nil
S14	24	M	Right	Nil

**Table 2:** Classification accuracies offered by different SCP features.

Sl. No.	Binary Pair	Classification Accuracy (%)	
		Direction-Speed	Speed
1	Mean SCP	58.78 ± 8.7	52.53 ± 9.7
2	Peak negativity	63.44 ± 9	53.87 ± 6.4
3	Peak negativity latency	55.8 ± 5.3	53.72 ± 9.4
4	All the three features	61.95 ± 7.6	55.2 ± 9.7

**Table 3:** Classification accuracies offered by MI EEG and movement observation EEG.

Binary Pair	Classification Accuracy (%)		Channel Size		*p*-Value
	MI EEG	Mirror EEG	MI EEG	Mirror EEG	
Left Slow/Left Fast	62.2 ± 14.4	52.98 ± 7.6	5.2 ± 1.8	3.5 ± 1.5	0.12
Left Slow/Right-Slow	62.8 ± 11.9	58.63 ± 15.4	5.4 ± 1.7	4.8 ± 1.8	0.21
Left Slow/Right Fast	64.58 ± 16	63.39 ± 13.9	5.4 ± 2.1	5.2 ± 1.7	0.84
Left Fast/Right-Slow	62.8 ± 14.2	62.2 ± 16.9	5.2 ± 1.8	4.5 ± 1.9	0.96
Left Fast/Right Fast	71.13 ± 15.2	61.31 ± 16.6	5.1 ± 1.7	4.5 ± 1.8	0.12
Right-Slow/Right Fast	57.14 ± 10.5	47.92 ± 8.9	5.3 ± 1.1	5.3 ± 1.1	0.12
Mean	63.44 ± 9	57.74 ± 8.6	5.3 ± 0.9	4.6 ± 0.8	-
Slow/Fast	53.87 ± 6.4	50.74 ± 8.1	4.4 ± 2	5.3 ± 1.9	0.44

**Table 4:** Peak value of Pearson correlation coefficient between SCP of MI and OB EEG segments.

Subject Id	Left-Slow		Left-Fast		Right-Slow		Right Fast	
	PCC	Channel	PCC	Channel	PCC	Channel	PCC	Channel
S1	0.7	FCz	0.76	CP2	0.95	FC2	0.57	C3
S2	0.88	FC1	0.83	FC2	0.96	Cz	0.86	FC1
S3	0.94	FC4	0.87	CP1	0.92	C4	0.83	CP3
S4	0.97	FC3	0.54	C3	0.81	C4	0.84	FC2
S5	0.8	FCz	0.7	CP3	0.78	CPz	0.68	C4
S6	0.77	FC4	0.84	C4	0.18	CP3	0.58	CP2
S7	0.67	FC4	0.67	Cz	0.47	C1	0.48	FC1
S8	0.67	FC4	0.85	Cz	0.57	C1	0.75	C1
S9	0.85	CPz	0.95	C4	0.54	CP4	0.81	FC1
S10	0.72	CP3	0.87	FC1	0.78	FC3	0.87	CP2
S11	0.63	CP2	0.83	C2	0.26	FC3	0.54	FCz
S12	0.64	CPz	0.65	CPz	0.64	CP2	0.73	C3
S13	0.79	Cz	0.9	FC2	0.91	FC2	0.96	CP1
S14	0.88	C3	0.73	C2	0.62	CP4	0.74	CP3

**Table 5:** Performance comparison with existing method.

Method	Average Classification Accuracy (%)			
	Direction-Speed		Speed	
	MI	OB	MI	OB
Proposed Method	63.44 ± 9	57.74 ± 8.6	53.87 ± 6.4	50.74 ± 8.1
WCSP + MRCP [18]	65.22 ± 9.8	61.01 ± 9.1	54.61 ± 9.9	53.27 ± 7.9

## Data Availability

The datasets used in the current study are not publicly available due to privacy/ethical restrictions but are available from the corresponding author on reasonable request.

## Abbreviations

The following abbreviations are used in this manuscript:

MI-BCI	Motor imagery-based brain-computer interface
EEG	Electroencephalogram
SCP	Slow cortical potential
BCI	Brain-computer interface
MRCP	Movement-related cortical potential
WCSP	Wavelet common spatial pattern
PCC	Pearson correlation coefficient
